# Relationships between Body Fat Distribution, Epicardial Fat and Obstructive Sleep Apnea in Obese Patients with and without Metabolic Syndrome

**DOI:** 10.1371/journal.pone.0047059

**Published:** 2012-10-08

**Authors:** Carla Lubrano, Maurizio Saponara, Giuseppe Barbaro, Palma Specchia, Eliana Addessi, Daniela Costantini, Marta Tenuta, Gabriella Di Lorenzo, Giuseppe Genovesi, Lorenzo M. Donini, Andrea Lenzi, Lucio Gnessi

**Affiliations:** 1 Department of Experimental Medicine, Section of Medical Physiopathology, Endocrinology and Food Science, University of Rome “Sapienza”, Rome, Italy; 2 Head and Neck Department, University of Rome “Sapienza”, Rome, Italy; Ochsner Health System, United States of America

## Abstract

**Background:**

Obstructive sleep apnea (OSA) and metabolic syndrome, both closely related to obesity, often coexist in affected individuals; however, body mass index is not an accurate indicator of body fat and thus is not a good predictor of OSA and other comorbidities. The aim of this study was to investigate whether the occurrence of OSA could be associated with an altered body fat distribution and a more evident cardio metabolic risk independently from obesity and metabolic syndrome.

**Methods and Results:**

171 consecutive patients (58 men and 113 women) were included in the study and underwent overnight polysomnography. Anthropometric data, blood pressure, lipid profile, glycaemic parameters were recorded. Body composition by DXA, two-dimensional echocardiography and carotid intima/media thickness measurement were performed. 67 patients (39.2%) had no OSA and 104 (60.8%) had OSA. The percentage of patients with metabolic syndrome was significantly higher among OSA patients (65.4%) that were older, heavier and showed a bigger and fatter heart compared to the control group. Upper body fat deposition index , the ratio between upper body fat (head, arms and trunk fat in kilograms) and lower body fat (legs fat in kilograms), was significantly increased in the OSA patients and significantly related to epicardial fat thickness. In patients with metabolic syndrome, multivariate regression analyses showed that upper body fat deposition index and epicardial fat showed the best association with OSA.

**Conclusion:**

The occurrence of OSA in obese people is more closely related to cardiac adiposity and to abnormal fat distribution rather than to the absolute amount of adipose tissue. In patients with metabolic syndrome the severity of OSA is associated with increase in left ventricular mass and carotid intima/media thickness.

## Introduction

Obesity is a common finding and a major pathogenetic factor in obstructive sleep apnea (OSA) [1]. OSA is characterized by recurrent episodes of absent or decreased airflow in the upper airway during sleep and most often arises in obese individuals who have a narrowing of the upper airway because of fatty deposits in the tongue and para-pharyngeal areas. Intermittent hypoxia, sleep fragmentation and increased cardiovascular risk are conditions associated with OSA [2], [3]. Obesity is a risk factor for diabetes and cardiovascular events [4], [5]. Adipocytes and inflammatory cells show a high degree of interaction in obesity and exert important endocrine functions, involving multiple cross talks with other tissues and different fat depots in the body [6], [7]. Body mass index (BMI) is not an accurate indicator of body fat and thus is not a good predictor of comorbidities [8]. Most adult patients with OSA have central obesity and increased visceral fat [9], the latter being associated with neck adiposity, increased upper airway fat [10] and metabolic abnormalities [11]. Gender-related differences in the amount of visceral fat [12], [13] could contribute to the higher prevalence of OSA in men. In recent years a number of studies have suggested a strong bidirectional association between OSA and metabolic syndrome (MetS), the commonly used term for the clustering of cardio metabolic risk factors including visceral obesity, hypertension, dyslipidaemia and type 2 diabetes mellitus [14]. The prevalence of MetS varies from 74 to 85% among patients with OSA [15]. Interestingly, it has been recently demonstrated that continuous positive airway pressure therapy lowers blood pressure and partially reverses metabolic abnormalities in MetS patients [16], further emphasizing the relationship between OSA and MetS.

## Methods

### Objective

The aim of this study was to investigate whether the presence and severity of OSA associates with cardiovascular functional and structural changes and a more compromised metabolic phenotype than obesity and MetS per se by evaluating polysomnographic records, body fat distribution, echocardiographic findings and cardio metabolic risk factors in obese patients either healthy or affected by MetS.

### Participants

171 consecutive obese patients of Caucasian origin (58 men and 113 women) were included in the study. All participants were asymptomatic outpatients admitted for routine check-up evaluations and underwent a detailed history, physical examination and overnight polysomnography at a hospital-based sleep laboratory [17]. Exclusion criteria were the presence of overt endocrinopathy, acute illnesses, heart diseases, and any respiratory disorder other than OSA, uncontrolled hypertension, craniofacial abnormalities, smoking status, current use of hypnotics or any treatment for breathing disorders.

### Ethics

All subjects were enrolled after written consent and approval by the Ethic Committee of Sapienza, University of Rome, Italy.

### Description of procedures

#### Metabolic characterization

Metabolic characterization included: anthropometric measurements [weight, height, waist circumference (WC), hip circumference (HC)], measurement of systolic and diastolic blood pressure (BP) and hearth rate (HR), lipid profile [triglycerides, total cholesterol (TOT-C), low-density lipoprotein cholesterol (LDL-C), and high-density lipoprotein cholesterol (HDL-C)], glycaemic parameters [fasting plasma glucose (FPG), HbA1c], and fasting insulin. BP was measured twice in the sitting position after 5 min of rest (Omron-5M automatic device). Obesity was defined as body mass index (BMI) ≥30 kg/m2. Waist/hip ratio (WHR) was determined by measuring the WC with soft tape on standing subjects just above the iliac crest and the HC in a horizontal plane at the level of the maximal extension of the buttocks. All laboratory specimens were drawn after a 12-h fasting period. FPG determinations were performed using the hexokinase method (Aeroset, Abbott Park, IL, USA). HbA1c was measured by the Variant II HbA1c analyzer based on chromatographic separation on a cation-exchange cartridge (Bio-Rad Laboratories, Hercules, CA). Plasma insulin was measured using electrochemiluminescence immunoassay (Roche Modular E170 analyzer; Roche Diagnostics GmbH, Mannheim, Germany). TOT-C, HDL-C, and triglyceride concentrations were measured by standard enzymatic methods using Roche Diagnostic's reagents with an automated analyzer (Roche Modular P800). LDL-cholesterol was calculated by using Friedewald's equation [18]. Homeostasis model assessment of insulin resistance (HOMA-IR) was calculated from Matthews et al [19]. All samples were assayed in duplicate with intrassay and interassay mean coefficients of variation of 2% and 4%, respectively.

#### Metabolic Syndrome

The MetS was defined according to the National Cholesterol Education Program's Adult Treatment Panel III criteria, with the cut-off value for defining abdominal obesity of 94 cm for men and 80 cm for women [14].

#### Dual energy X-ray absorptiometry (DXA)

DXA analysis was performed by one single experienced technician using a DXA scan (Hologic Inc., Bedford, MA, USA, QDR 4500W). Coefficient of variation for fat mass was <1.5%. Body composition was measured in the whole body and in specific body regions. Delimiters for regional analysis were determined by standard software (Hologic Inc., S/N 47168 VER. 11.2). With the use of specific anatomic landmarks, regions of the head, trunk, arms and legs were distinguished. Scans were performed according to the manufacturer's instructions [20], [21].

#### Polysomnography

Polysomnography was performed overnight between 10:00 pm and 6:00 am. Polysomnographic records were scored according to standard criteria [22]. Apnea was defined by an 80% or greater reduction in the airflow signal with persistent respiratory effort lasting 10 seconds or longer. Hypopnea was defined as a 30% or greater reduction in the airflow signal with persistent respiratory effort lasting at least 10 seconds associated with a desaturation of 4% or greater. OSA degree was determined by the apnea/hypopnea index (AHI) defined as the total number of obstructive apneas and hypopneas per hour of sleep. Absence of OSA or mild, moderate and severe degrees were defined by an AHI of ≤4.9, 5-14.9, 15-29.9, and ≥30 events/hour, respectively.

#### Echocardiography

Participants underwent high-resolution M-B mode transthoracic echocardiography using a 2.5 MHz Probe (Esaote MyLab40, Esaote Europe B.V., The Netherlands). Two-dimensional echocardiography and standard M-mode measurements of left ventricle were performed, according to the recommendations of the American Society of Echocardiography [23]. Left ventricular mass index (LVMI) was recorded. The epicardial fat thickness (EFT) was identified as the echo-free space between the outer wall of the myocardium and the visceral layer of the pericardium, and its thickness was measured perpendicularly on the free wall of the right ventricle at end-systole in three cardiac cycles. Parasternal long- and short-axis views were used. The average value of three cardiac cycles from each echocardiographic view was considered [24], [25]. The inter- and intra-observer coefficient of variation was 2%.

#### Carotid intima/media thickness (cIMT) measurement

Spectral Doppler exam of the common carotid artery was performed with a 7.5 MHz probe. The cIMT was measured in the anterior and posterior wall of the common carotid artery as the distance from the trailing edge of the adventitia to the leading edge of the intima-media. The cIMT values for any given subject were the mean value for the two common carotid arteries. All echocardiograms and carotid ultrasonographies were recorded by the same experienced operator who was blinded to the other study data.

#### Statistical methods

Data were analyzed with the use of STATISTICA software, version 8.0 (Stat Soft, Inc., Tulsa, Oklahoma). Results are expressed as mean±SD. Differences between groups were analyzed using ANOVA for continuous variables. Pearson correlation test was used to measure a linear association between variables. The roles of sex, age, BMI, body fat distribution, cIMT, EFT, glucose and serum lipids as associated variables with AHI were tested by linear regression with the use of multivariate models. All P values presented are two-tailed, and values less than 0.05 are considered statistically significant.

## Results

The baseline characteristics of the subjects studied are listed in *Table 1*. 67 patients (39.2%) had no OSA. Among the 104 apneic patients, 42 (24.6%) presented mild OSA, 26 (15.2%) and 36 patients (21.0%), moderate and severe OSA, respectively. Compared to control group, patients with OSA were older, heavier, had higher BP, BMI, WC, HC, WHR, HbA1c, FPG, fasting insulin, HOMA-IR and triglycerides.

**Table 1:** Baseline Characteristics of the Study Population, according to the severity of sleep disordered breathing.

	All (n = 171)	No OSA (n = 67)	Mild OSA (n = 42)	Moderate OSA (n = 26)	Severe OSA (n36)
Gender (F/M)	113/58	51/16	31/11	15/11	16/20
AHI (events/h)	27.03±22.19	1.54±1.61	8.90±2.82^c^	20.79±4.04^c^	53.23±17.37^c^
Age (years)	46.95±13.32	41.97±13.36	46,00±12,48	55.81±12.27^c^	50.94±10.62^c^
Weight (Kg)	112.49±32.32	99.12±23.27	110,81±30.39^a^	116.94±25.89^c^	136.09±39.50^c^
BMI (Kg/m^2^)	40.39±9.25	35.70±6.48	41.08±9.08^c^	42.23±7.17^c^	47.01±10.6^c^
WC (cm)	122.16±19.44	111.47±16.27	119.65±13.7^c^	130.76±14.7^c^	139.14±19.81^c^
HC (cm)	124.19±14.13	121.90±14.82	119.17±15.99	130.00±8.77	131.50±12.93
WHR	0.98±0.09	0.96±0.06	0.97±0.14	1.015±0.08^a^	0.98±0.078^a^
HbA1c (%)	5.98±0.98	5.55±0.52	5.99±0.76^b^	6.52±1.50^c^	6.34±1.04^c^
FPG (mmol/L)	5.67±1.64	5.21±1	5.6±1.08^a^	6.45±3.07^b^	5.96±1.45^c^
Insulin (pmol/L)	211.82±137.44	165.01±96.67	214.81±143.34^a^	216.34±147.79	288.01±154.46^c^
HOMA-IR	8.16±7.21	5.61±3.69	7.93±5.39^b^	9.97±10.05^c^	11.64±9.52^c^
Systolic BP (mmHg)	128.42±15.63	122.67±15.96	126.86±14.26	135.40±14.71^c^	135.55±12.52^c^
Diastolic BP (mmHg)	81.55±9.75	77.91±9.58	80.48±8.89	85.20±9.41	86.47±8.59^c^
TOT-C (mmol/L)	5.11±1.20	4.92±1.35	5.02±0.96	5.23±1.22	5.46±1.1^a^
LDL-C (mmol/L)	3.24±1.01	3.20±1.10	3.12±0.85	3.22±1.04	3.47±0.99
HDL-C (mmol/L)	1.23±0.33	1.25±0.36	1.23±0.38	1.22±0.26	1.21±0.23
Triglycerides (mmol/L)	1.56±0.97	1.29±0.59	1.61±1.14	1.75±1.31^a^	1.84±0.96^c^
TOT-C/HDL ratio	4.36±1.28	4.09±1.13	4.41±1.23	4.49±1.64	4.69±1.28^a^
Triglycerides/HDL ratio	3.21±2.59	2.65±1.88	3.38±2.80	3.73±4.08	3.67±2.04^a^
MetS n (%) #	92 (53.8)	24 (35.8)	23 (44.8%)	18 (69.2%)	31 (86.1%)
MetS criteria without WC	1.69±1.10	1.27±0.99	1.71±1.29^a^	2.15±0.92^c^	2.28±0.70^c^

Values represent mean±standard deviation unless otherwise indicated.

#Number of patients with MetS. Percentage values in parentheses.

a p<0.05 vs. no OSA.

b p<0.01 vs no OSA.

c p<0.001 vs no OSA.

*Table 2* shows the body composition data according to the severity of sleep disordered breathing. The percentage of fat mass was almost superimposable between the control and OSA groups, with the notable exception of the head and trunk fat and Upper body Fat Deposition Index (UFDI), the ratio between upper body fat (head, arms and trunk fat in kilograms) and lower body fat (legs fat in kilograms), that were significantly higher in the apneic patients.

**Table 2:** Body composition (upper) echocardiographic findings and cIMT measurements (lower) according to the severity of sleep disordered breathing.

	All (n = 155)	No OSA (n = 66)	Mild OSA (n = 33)	Moderate OSA (n 24)	Severe OSA (n32)
Body Fat (%)	38.22±7.16	37.78±7.22	38.88±8.35	38.08±6.26	38.59±6.22
Head fat (%)	20.34±1.67	20.81±1.54	21.21±1.57	21.80±0.87^b^	22.43±2.17^c^
Trunk Fat (%)	38.42±7.43	37.12±7.11	39.49±9.05	37.90±5.91	40.42±6.18
Left arm fat (%)	46.73±10.33	45.09±9.66	48.47±11.75	47.53±8.94	49.05±10.65
Right arm fat (%)	45.32±10.84	43.46±10.21	46.92±12.17	45.49±9.77	47.90±11.25
Left leg fat (%)	39.20±8.57	39.70±7.89	39.84±9.46	39.27±8.83	36.77±8.88
Right leg fat (%)	40.18±8.66	40.51±8.21	40.54±9.35	40.31±9.23	38.60±8.65
UFDI	1.91±0.64	1.75±0.44	1.97±0.63^a^	1.81±0.65	2.36±0.88^c^
	**(n 171)**	**(n 67)**	**(n 42)**	**(n 26)**	**(n 36)**
EFT (mm)	8.41±1.62	7.62±0.92	8.23±1.12^b^	8.75±0.85^c^	9.23±1.39^c^
LVMI (g/m^2^)	112.44±22.45	105.20±22.56	110.00±17.32	124.68±19.04^c^	121.32±25.15^b^
c IMT (mm)	0.77±0.18	0.71±0.16	0.77±0.14	0.78±0.19	0.85±0.22^b^

Values represent mean±standard deviation unless otherwise indicated.

a p<0.05 vs no OSA.

b p<0.01 vs no OSA.

c p<0.001 vs. no OSA.

As expected, OSA patients showed a bigger and fatter heart and an increased cIMT compared to the control group.

**Figure 1:**
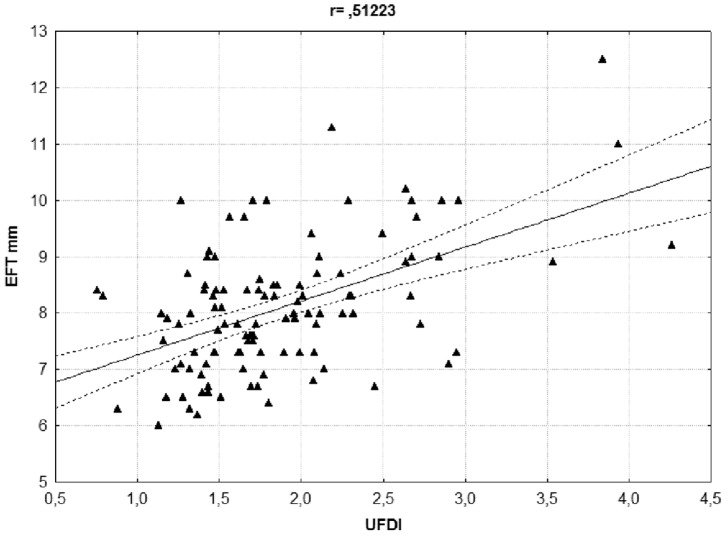
Linear regression analysis between epicardial fat thickness (EFT) and upper body fat deposition index (UFDI). r = 0.51223; p<0.001

The measures of EFT and of UFDI showed close co-linearity and were significantly related (*Figure 1*).

**Figure 2:**
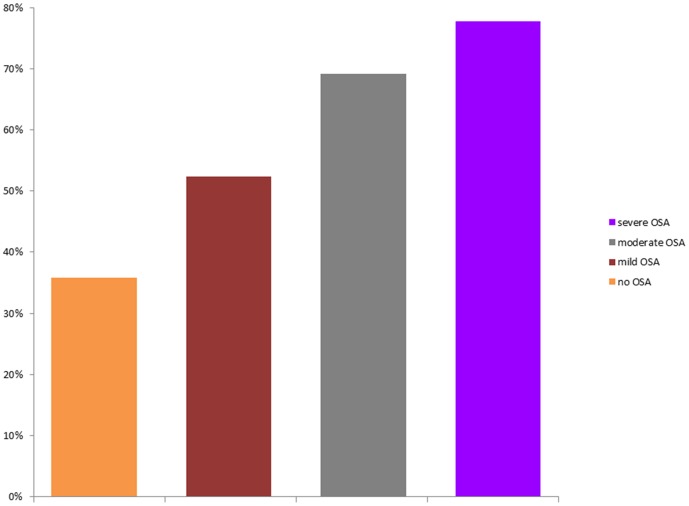
Percentage of patients with metabolic syndrome (MetS) according to sleep disordered breathing.

The percentage of patients with MetS was significantly higher among apneic patients compared with control ones (65.4% vs. 35.8%, respectively; p<0.001) and increased with the severity of OSA: 52.4% of the patients with mild OSA (22/42), 69.2% of the subjects with moderate OSA (18/26) and 77.8% of the patients with severe OSA (28/36) had MetS (*Figure 2*).

We then considered only MetS patients (92/171) and compared control and OSA patients within this group (*Table 3*). OSA patients were once again heavier but not fatter, showing a particular fat distribution pattern characterized by a significantly greater UFDI than control group. Systolic and diastolic BP values were higher in apneic patients that were also more insulin-resistant and showed higher levels of HbA1c than control ones. Increased ILVM, EFT and cIMT were present in MetS OSA patients compared with controls.

**Table 3:** Characteristics of patients with metabolic syndrome according to the severity of sleep disordered breathing.

Parameters	No OSA (n = 23)	OSA (n = 67)	Mild OSA (n = 22)	Moderate OSA (n = 18)	Severe OSA (n = 27)
AHI (events/h)	1.47±1.46	30.32±22.12	8.83±2.91^a^	20.483.75^c^	51.98±15.72^c^
Gender (F/M)	17/6	41/26	17/5	10/8	14/13
Age (years)	44.71±15.18	50.46±12.05	47.74±13.88	54.61±10.91^a^	50.06±10.89
BMI (kg/m^2^)	36.72±5.64	44.23±9.54^c^	40.68±7.65^a^	42.01±6.48^b^	48.14±10.97^c^
WC (cm)	113.39±20.75	131.05±18.19^c^	121.35±12.67	129.17±12.82^b^	140.24±20.71^c^
HC (cm)	123.43±12.92	127.75±12.15	123.70±18.16	128.78±7.24	130.50±14.71
WHR	0.96±0.08	1.02±0.13^a^	1.01±0.22	1.025±0.09	1.005±0.06
Head fat (%)	21.31±1.53	21.68±1.76	20.72±1.28	21.64±0.80	22.73±2.30^a^
Body Fat (%)	36.26±7.67	38.64±7.18	40.14±8.42	36.93±6.07	38.59±6.69
Trunk Fat (%)	36.11±7.35	39.58±7.38^a^	41.02±9.47	36.72±5.11	40.62±6.65
Left arm fat (%)	44.06±8.08	48.35±9.03^a^	49.38±11.63	46.23±9.43	50.04±11.07^a^
Right arm fat (%)	42.06±9.80	46.56±11.48^b^	47.78±12.62	44.10±10.00	49.26±11.58^a^
Left leg fat (%)	36.99±7.16	37.92±9.91	40.21±10.26	37.82±8.95	36.22±9.56
Right leg fat (%)	38.09±7.88	39.15±9.71	40.94±9.73	38.97±9.44	38.03±9.32
UFDI	1.85±0.32	2.14±0.53^b^	2.07±0.70	1.86±0.70	2.51±0.90^b^
Systolic BP (mmHg)	129.69±16.50	136.34±12.72^a^	134.78±10.39	138.89±14.10	136.13±13.08
Diastolic BP (mmHg)	80.22±10.82	85.49±8.48^a^	83.70±8.15	86.94±8.77^a^	86.71±9.07
TOT-C (mmol/L)	4.81±1.29	5.21±1.13	4.81±0.9	5.43±1.32	5.46±1.11^a^
LDL-C (mmol/L)	3.15±1.03	3.23±0.97	2.77±0.64	3.43±1.10	3.44±0.99
HDL-C (mmol/L)	1.09±0.28	1.12±0.23	1.07±0.26	1.15±0.24	1.18±0.24
Triglycerides (mmol/L)	1.7±0.75	1.97±1.26	1.99±1.4	1.98±1.52	1.94±0.99
FPG (mmol/L)	5.72±1.29	6.40±2.21	6.2±1.11	6.97±3.58	6.03±1.55
Insulin (pmol/L)	166.61±105.91	267.45±160.22^b^	264.79±171.33^b^	258.03±176.02	305.06±164.62^b^
HOMA-IR	6.31±4.66	11.50±9.42^b^	9.99±5.97^a^	12.46±11.69^a^	12.05±10.00^a^
Hba1c (%)	5.79±0.54	6.52±1.25^b^	6.22±0.91	6.85±1.64^b^	6.41±1.07^a^
EFT (mm)	8.22±0.97	8.97±1.13^b^	8.38±1.00	8.83±0.88	9.40±1.41^b^
ILVM (g/m^2^)	111.80±20.80	119.68±22.75	110.80±16.89	124.08±18.52	123.88±22.17^a^
cIMT (mm)	0.74±0.22	0.79±0.18	0.76±0.13	0.76±0.20	0.84±0.12^a^

Values represent mean±standard deviation unless otherwise indicated.

a p<0.05 vs no OSA.

b p<0.01 vs no OSA.

c p<0.001 vs. no OSA.

The presence of a strong correlation between EFT and UFDI prevents any attempt to compare the effect of these variables in the same regression model. Two distinct multivariate regression analyses, adjusted for age and sex, were performed in patients with MetS to determine whether obesity, as expressed by BMI, or parameters of local fat distribution (WC, HC, WHR, UFDI) primarily correlate with the severity of OSA (*Table 4*) and which cardiovascular parameter best associates with AHI (*Table 5*). UFDI, rather than BMI, i.e. altered fat distribution rather than obesity per se and EFT, a marker of cardiac steatosis, were the best predictors of OSA.

**Table 4:** Evaluation of effects of age, sex, BMI, WC, HC, WHR and UFDI on AHI with multivariate regression analysis in patients with metabolic syndrome (adjusted R^2^ = 52483790; p<00002).

Parameters	β	ES	p value
Age	−0.18624	0.758620	0.808453
Sex	0.40259	0.530958	0.456732
BMI	3.47049	2.170903	0.124839
WC	−2.94049	2.318892	0.218656
HC	−0.61747	1.547565	0.694126
WHR	−3.13552	1.718780	0.083879
UFDI	0.73746	0.137864	0.000017

**Table 5:** Evaluation of effects of age, sex, BMI, EFT, LVMI, cIMT on AHI with multivariate regression analysis in patients with metabolic syndrome (adjusted R^2^ = 59617544 p<.00000).

Parameters	β	ES	p value
Age	0.421418	0.580703	0.473836
Sex	0.177947	0.509540	0.729439
BMI	0.136449	0.735003	0.854016
cIMT	−0.337980	0.610544	0.584114
ILVM	−0.664158	0.822765	0.426107
EFT	0.762926	0.1109871	0.000000

## Discussion

OSA is frequently associated with obesity [26]. However, It is well known that BMI is not a good measure of body adiposity [8] and different factors beyond BMI are associated with OSA, with abdominal fat, gender and age being significant predictors of sleep disordered breathing [27], [28].

Sleep disordered breathing was identified as an independent, dose-dependent risk factor for hypertension [29] and for insulin resistance and diabetes development [30]; untreated severe OSA independently increases the odds of fatal and nonfatal cardiovascular events [31]. OSA and MetS, both closely related to obesity, often coexist in affected individuals [32]. Accordingly, the parameters related to MetS, such as triglycerides, BP, FBG, WC, and other cardio metabolic risk factors, such as HbA1C, fasting insulin levels, HOMA-IR, were significantly increased in apneic patients and the prevalence of MetS was significantly higher in the severe OSA group (Figure 2).

It is thought that, in obese individuals, fat deposits in any part of the upper airway, increasing the total volume of soft tissue within the maxillomandibular enclosure, narrow the pharynx and increase the upper airways collapsibility [33], thereby predisposing to OSA. In addition, intramuscular fat content in the posterior tongue is significantly increased in obese patients and rat fed a high fat diet show an increase of the percentage of oil droplet areas in the genioglossus and geniohyoid muscles [34], [35]. Interestingly, the amount of adipose tissue adjacent to the pharyngeal airway and in the intra peritoneal space directly associates with AHI, but not with BMI [36]. These data reinforce the assumption that BMI is not a good predictor of OSA, whether abdominal fat and truncal obesity indices are more sensitive parameters for prediction [27], [37].

Various measures of fat distribution by DXA can predict insulin resistance and MetS [38]. We found a specific pattern of adiposity in OSA patients consisting in increased arms, trunk and head fat in the presence of superimposable legs adipose tissue content. This is the first time to our knowledge that the arms adipose tissue content is included in the evaluation of central obesity, although an increased arms fat content in post-menopausal women and in Cushing syndrome has been found but never associated with metabolic derangement [39], [40]; UFDI strictly correlates with both AHI and EFT suggesting that this new parameter might have the property to highlight in obese patients the risk to develop OSA and cardiovascular diseases.

Sleep-disordered breathing is prevalent among the population with heart failure and preserved LVEF [41], [42] and can impair LV diastolic function [43]. Furthermore, a link between OSA and atherosclerosis has been demonstrated [44]. EFT strongly and independently reflects the intra-abdominal visceral fat as measured by magnetic resonance imaging [24] and intra-myocardial lipid content, as measured by proton magnetic resonance spectroscopy [45]. A growing number of studies indicate that EFT measurement may play a role in the stratification of the cardio-metabolic risk [46] and that is also significantly and independently related to MetS and other traditional cardiovascular risk factors [47].

In our patients, echocardiographic evaluation showed a bigger and fatter heart: LVMI and EFT were significantly increased in severe OSA group, suggesting a worsening of cardiac structural changes in relation to AHI progression. OSA patients also showed an increased cIMT, a marker of subclinical atherosclerotic disease.

A significant number of our patients had both OSA and MetS, a condition that has been defined as a new pathological entity termed syndrome Z [48]. We found that patients with syndrome Z, although had a higher BMI, were not fatter than MetS patients without OSA and showed a clear alteration in body fat distribution. In fact, supporting the data of *McLaughlin T et al*. [49], the UFDI values were higher in these patients who had also higher levels of HbA1C and were more insulin-resistant. Furthermore, OSA patients with MetS showed higher BP, more pronounced heart modifications and an increased cIMT. Thus, OSA severity seems to worsen the cardio metabolic risk expected from MetS *per se*.

EFT and UFDI were analyzed separately by multiple regression analysis because, being the 2 parameters highly correlated, they both convey essentially the same information and neither may contribute significantly to the model after the other one is included. The multivariate regression analysis performed only in patients with MetS and involving BMI and fat distribution parameters (*Table 4*) showed that UFDI was the only parameter significantly associated with AHI. A further model designed to evaluate which cardiovascular parameter showed the best association with AHI, revealed that EFT, a marker of fat storage in myocardial tissue, was the most reliable (*Table 5*).

Considering that echocardiography is safer than a radiation involving technique like DXA, and that UFDI and EFT can provide the same information on the association with OSA severity, we suggest to measure EFT in patients with Mets.

It is well known that hepatic, epicardial, skeletal and myocardial muscle fat accumulation increases cardio metabolic risk [50]. Ectopic fat deposition associates with insulin resistance and mitochondrial defects [51], [52] and occurs when subcutaneous adipose tissue is unable to store energy excess [53]. In our patients, UFDI directly associates with both AHI, a parameter related to the amount of adipose tissue adjacent to the upper airway and EFT, a measure of cardiac ectopic fat. Thus UFDI could be an indirect index of ectopic fat deposition and of reduced subcutaneous fat accumulation. Altered lipid partitioning within muscle and myocardial triglyceride stores were independently associated with carotid atherosclerosis, insulin resistance and type 2 diabetes [54], [55]. Interestingly, HbA1c levels and cIMT were significantly increased in the patients affected by both MetS and OSA, suggesting the occurrence of muscular and myocardial steatosis in this group.

###  Limitations

Our study has several limitations. First we considered only obese patients and the study groups were not homogeneous in terms of gender distribution. Our sample had also a limited racial background. In addition the study was cross sectional so we cannot infer a causal link between the parameters evaluated and OSA. A prospective study involving therapeutic interventions, like CPAP or diet induced weight loss, is required to assess the causal relationship between modifications of fat distribution, epicardial fat and AHI.

### Conclusions

The major strength of this study is the highlighting of the close relationship between UFDI and echocardiographic abnormalities with sleep disordered breathing in obese patients.

In conclusion, the occurrence of OSA in obese people relates to abnormal fat distribution and to EFT, rather than to the amount of adipose tissue per se, and the presence and severity of OSA seem to worsen the cardio metabolic risk already established by MetS. The phenotype of obesity characterized by increased UFDI may be suggestive on one side of OSA and on the other can reflect cardiac structural changes and adiposity. Myocardial steatosis, as measured by EFT, may mirror the ectopic fat deposition in upper airway muscles that plays an important role in disordered breathing.
